# Urinary Sodium and Potassium Excretion and Dietary Sources of Sodium in Maputo, Mozambique

**DOI:** 10.3390/nu9080830

**Published:** 2017-08-03

**Authors:** Ana Queiroz, Albertino Damasceno, Neusa Jessen, Célia Novela, Pedro Moreira, Nuno Lunet, Patrícia Padrão

**Affiliations:** 1 Faculdade de Ciências da Nutrição e Alimentação da Universidade do Porto, Rua Dr. Roberto Frias, 4200-465 Porto, Portugal; anaqueiroz91@hotmail.com (A.Q.); pedromoreira@fcna.up.pt (P.M.); 2 Faculdade de Medicina da Universidade Eduardo Mondlane, Avenida Salvador Allende, n° 702, 1111 Maputo, Mozambique; tino_7117@hotmail.com (A.D.); neusa.jessen@gmail.com (N.J.); celianovela@gmail.com (C.N.); 3 Departamento de Ciências da Saúde Pública e Forenses e Educação Médica, Faculdade de Medicina da Universidade do Porto, Alameda Prof. Hernâni Monteiro, 4200-319 Porto, Portugal; nlunet@med.up.pt; 4 EPIUnit-Instituto de Saúde Pública, Universidade do Porto, Rua das Taipas, n° 135, 4050-600 Porto, Portugal; 5 Centro de Investigação em Atividade Física, Saúde e Lazer, Universidade do Porto, Rua Dr. Plácido da Costa, 4200-450 Porto, Portugal

**Keywords:** sodium, salt, urinary sodium, urinary potassium, Mozambique, Africa

## Abstract

This study aimed to evaluate the urinary excretion of sodium and potassium, and to estimate the main food sources of sodium in Maputo dwellers. A cross-sectional evaluation of a sample of 100 hospital workers was conducted between October 2012 and May 2013. Sodium and potassium urinary excretion was assessed in a 24-h urine sample; creatinine excretion was used to exclude unlikely urine values. Food intake in the same period of urine collection was assessed using a 24-h dietary recall. The Food Processor Plus^®^ was used to estimate sodium intake corresponding to naturally occurring sodium and sodium added to processed foods (non-discretionary sodium). Salt added during culinary preparations (discretionary sodium) was computed as the difference between urinary sodium excretion and non-discretionary sodium. The mean (standard deviation) urinary sodium excretion was 4220 (1830) mg/day, and 92% of the participants were above the World Health Organization (WHO) recommendations. Discretionary sodium contributed 60.1% of total dietary sodium intake, followed by sodium from processed foods (29.0%) and naturally occurring sodium (10.9%). The mean (standard deviation) urinary potassium excretion was 1909 (778) mg/day, and 96% of the participants were below the WHO potassium intake recommendation. The mean (standard deviation) sodium to potassium molar ratio was 4.2 (2.4). Interventions to decrease sodium and increase potassium intake are needed in Mozambique.

## 1. Introduction

High sodium intake increases blood pressure (BP) and negatively affects endothelial and cardiovascular function, being positively associated with kidney disease, and cardiovascular morbidity and mortality [1,2,3]. Monitoring sodium intake at a population level, including the assessment of the contribution of different dietary sources of sodium to the overall consumption, are key aspects when designing interventions to control this risk factor.

The upper limit for sodium intake recommended by the World Health Organization (WHO) is two grams per day, corresponding to five grams of salt (sodium chloride)/day [4]. However, population-based data on sodium intake around the world shows that the intake far exceeds the recommendations [5]. In addition, potassium is another key nutrient that is inversely associated with blood pressure [6,7], and its relation with sodium intake should be taken into account when assessing the adequacy of sodium intake. Potassium increases urinary sodium excretion and reduces the risk of stroke and cardiovascular disease, attenuating sodium’s negative effects [8,9]. In fact, the effects of high sodium and low potassium intake on BP levels have been regarded as synergic [10,11,12] The sodium sensitivity of blood pressure and, consequently, the risk of hypertension, have been shown to increase with diets low in potassium [13] and, also of note, a higher intake of potassium has even more benefits for those with a high intake of sodium [14]. 

The WHO recommends a minimum daily intake of 3510 mg of potassium per day, and that the ratio of sodium to potassium (Na/K ratio) should be one to one, which should be achievable if the WHO guidelines for those nutrients are attained. Otherwise, if the levels of consumption of sodium are high, the recommended level of potassium intake must be increased in order to maintain the ratio at one [15]. Urinary Na/K ratio is considered an important measure, since it has been shown to represent a stronger marker of the relation of sodium and blood pressure [16]. Consequently, it is a better predictor of incident hypertension and of outcomes of blood pressure than the isolated urinary excretion of sodium or potassium, as reported in several studies [17,18,19,20], particularly in hypertensive adult populations [21].

Previous studies evaluating worldwide sodium and potassium intakes revealed overall high sodium and low potassium consumption [16], with a few regions, including some African populations, presenting low sodium and high potassium consumption [22]. In most of the Sub-Saharan Africa (SSA) countries, the intake of sodium has been shown to be well above that recommended by WHO [23].

Despite the lack of data on sodium and potassium intake in Mozambique, the monitoring of these exposures is of the utmost importance in this setting, given the high prevalence of hypertension (25–64 years: 33.1% in 2005 ) [24] and the increasing public health impact of cardiovascular diseases (CVD) in the country [25,26].

In the last decades, a steep increase in urbanization has been observed in Mozambique [27]. This will expectedly promote dietary changes, mostly involving decreases in the consumption of foods rich in potassium, such as legumes, fruits, vegetables, and a more frequent intake of processed foods, which often are energy dense and rich in salt [28]. A previous study on the culinary practices of Maputo inhabitants, conducted with a sub-sample of the present study, reported a frequent use of processed food products, such as sugar-sweetened beverages and sodium-rich powdered chicken stocks [29], reflecting the nutrition transition occurring in Mozambique [30,31]. 

We aimed to (i) evaluate the urinary excretion of sodium, potassium and sodium to potassium ratio and (ii) to estimate the contribution of discretionary (sodium from salt added during culinary preparations) and non-discretionary sodium (naturally occurring sodium and sodium added to processed foods) to the total sodium intake in a sample of Maputo inhabitants.

## 2. Materials and Methods

This is a cross-sectional study, based on a convenience sample of 100 adults, assembled between October 2012 and May 2013. Participants were selected among the workers of the Maputo Central Hospital. The sample included both lay workers and health professionals; all participants were Maputo dwellers, belonging to different households, aged 25 to 64 years. An incentive of 200 meticais (equivalent to around $4 United States dollar) was given to participants, to cover transportation costs and thus ensure participation. Demographic characteristics, including sex, age, and education, and a 24-h dietary recall were obtained in a face-to-face interview. Anthropometric measurements were taken and a 24-h urine sample was collected. 

### 2.1. 24-h Urine Collection

A container was supplied and participants were carefully instructed, through oral and written guidelines, to collect their urine over a 24-h period. They were taught to discard the first morning void and to collect all urine over the following 24 h, including the first void on the following morning, and to keep note of the time of the start and end of collection. This process occurred during weekdays and weekends, in periods not including any night shifts of the participants. Urine samples were analyzed for volume, creatinine, sodium and potassium. Sodium and potassium in urine were measured by flame photometry and creatinine by an automated validated enzymatic method.

To minimize systematic error due to incomplete urine collection, detailed instructions for a valid collection of urine were given orally and a leaflet was provided to each participant. In addition, a 3-L container was given to each participant to store the urine for 24 h, plus a 1-L plastic jug for each urine sample collection and a funnel to assist in both urine collection in the case of women, and in the transfer of urine from the jug to the 3-L container. Each participant was offered a backpack to facilitate the transportation of all this material when participants were away from home. Also, on the day of delivery of the 24-h urine participants were asked about the validity of their urine through the question, “How many times did you forget to pass urine sample in the counter during the 24 h”. Participants were also questioned about the occurrence of any problem that may have compromised the validity of urine.

The urinary creatinine excretion was used to exclude samples unlikely to represent a 24-h urine collection, either by undersampling or oversampling. We used the 24-h urinary creatinine excretion in relation to body weight, that is, creatinine coefficient = creatinine (mg/day)/weight (kg). Coefficients between 14.4 to 33.6 in men and 10.8 to 25.2 in women were considered sufficient to ensure that the samples corresponded to a 24-h period as recommended [32]. This led to the exclusion of the samples from 18 participants, and a total of 82 were considered for data analysis.

### 2.2. Dietary Intake

A 24-h dietary recall referring to the day of urine collection was obtained by a trained interviewer. Participants were asked to report all foods and beverages consumed in the reference period, aided by a photographic book and household measures (spoons, plates, cups and glasses) to quantify portion sizes. Data was collected regarding the amount of different foods consumed, identifying those consumed outside home, and also detailed information on the amount of added fat, sugar, chicken powdered stocks, salt added at the table and during cooking, and the use of other seasonings, the brand of processed foods, recipes and culinary methods. Food Processor Plus^®^ (Esha Research, Salem, OR, USA) was used to convert foods into nutrients; this software uses the U.S. Department of Agriculture food composition table, including raw and/or processed foods. Data referring to foods not available in the latter database was obtained from the Mozambican Food Composition Tables [33]. Data from the Brazilian Food Composition Table [34] was also used for foods not available in the Mozambican tables. For industrial food products, data from nutritional labels were used. Naturally occurring sodium and sodium added to processed foods (non-discretionary sodium), was then calculated and the salt added during culinary preparations (discretionary sodium) was estimated by the difference between urinary sodium excretion and non-discretionary sodium.

### 2.3. Anthropometric Measures

A SECA^®^ (Seca GmbH, Hamburg, Germany) digital scale with an embedded stadiometer was used for weight and height measurements, to the nearest 0.1 km and 0.1 cm, respectively. The participants were evaluated lightly clothed, barefooted, positioned in the center of the scale and with the head positioned in the Frankfort plan, according to standard procedures [35]. Body mass index (BMI) was calculated as the weight (kg) divided by square of height (m) and WHO cutoffs were used to define underweight (<18.5 kg/m^2^), normal weight (18.5–24.9 kg/m^2^), overweight (25.0–29.9 kg/m^2^) and obesity (≥30 kg/m^2^) [36].

A constant tension tape was used to measure waist circumference, directly over the skin at the level of the midpoint between the inferior margin of the last rib and the iliac crest in the mid-axillary-line, to the nearest 0.1 cm. Abdominal obesity was considered present when waist circumference was >88 cm for women and >102 cm for men [37].

### 2.4. Statistical Analysis

For comparisons between men and women, we used the following statistical tests: (i) the independent samples t-test for continuous socio-demographic, anthropometric and urinary parameters; (ii) the Mann–Whitney U test for dietary intakes; (iii) the Chi-Square for categorical variables. Data analysis was conducted using the Statistical Package for Social Sciences, version 23 (IBM Corporation, New York, NY, USA).

### 2.5. Ethics

The study protocol was approved by the Mozambican National Bioethics Committee for Health and written informed consent was obtained from all participants. The ethic approval code is 236/CNBS/12.

## 3. Results

The participants’ mean age was 40 years and approximately half reported more than primary school education. Just over half were classified as overweight or obese (Table 1).

As shown in Table 2, the most frequently consumed food groups in the previous 24 h (percentage of participants consuming, median intake among consumers) were cereal and cereal products (100%, 360 g), oils and fats (96%, 9 g) and vegetables (94%, 94 g).

A total of 90% of the participants consumed fruits and/or vegetables, from whom 26% met the recommended daily intake of at least 400 g [38].

Results from urine collection are shown in Table 3. Overall, the mean (standard deviation) urinary sodium excretion was 4220 (1830) mg/day and 92% of the participants did not meet the WHO recommendations for a maximum sodium intake of 2 g/day; in fact, almost half (56.4% of women and 41.9% of men) had a sodium intake above twice the recommended.

The mean (standard deviation) urinary potassium excretion was 1909 (778) mg/day, and 96% of the participants did not meet the WHO recommendations for minimum potassium intake. Mean (standard deviation) urinary sodium/potassium molar ratio was 4.7 (2.6) for women and 3.7 (2.1) for men (*p* = 0.06). 

**Figure 1:**
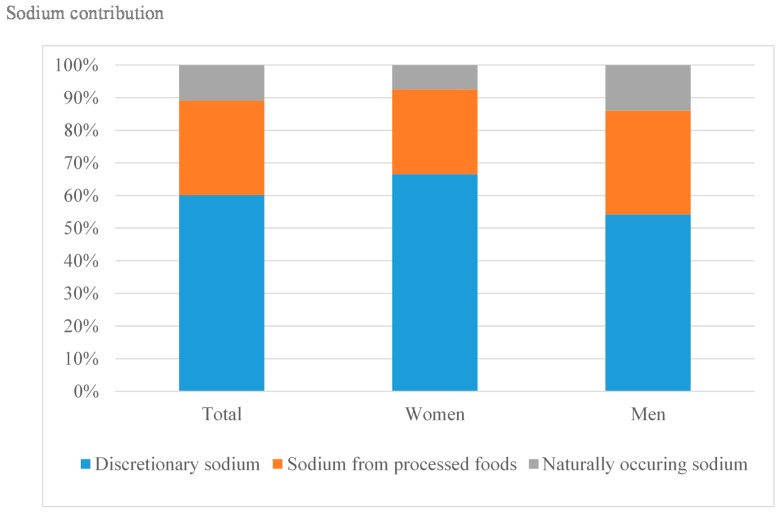
Mean sodium contribution (%) from discretionary salt use (added sodium during culinary preparations), salt added during processing (sodium from processed foods) and sodium intrinsic in food (naturally occurring sodium), overall and by sex.

Sodium from salt added during culinary preparations was the most important contributor to total sodium intake (all participants, 60.1%; women, 66.5%; men, 54.2%), followed by salt from processed foods (all participants, 29.0%; women, 26.0%; men, 31.8%). Naturally occurring sodium accounted for 10.9% of the overall intake (7.4% in women and 14.0% in men) (Figure 1).

In this sample, 69.5% of participants used chicken stocks for cooking or seasoning food, 96.3% added salt when cooking and 35.4% used salt for salad seasoning.

## 4. Discussion

To the best of our knowledge, this is the first study on sodium intake in the Mozambican population using 24-h urinary sodium excretion. Our results showed that nine out of every 10 participants exceeded the recommended sodium consumption, and the mean intake was more than twice the recommended by the WHO [4]. Sodium from salt added during culinary preparations accounted for almost two thirds of the total sodium intake.

Data on sodium intakes in African countries is scarce and most of the available studies are older than 15 years, many of them dating back over 30 years. This data was used in recently published systematic reviews that revealed sodium intakes in adult populations from African countries above the WHO recommended maximum of 2 g/day [23,39], with lower values found in Sub-Saharan Africa (<3.3 g/day) than in other world regions [39]. Nevertheless, a recent study conducted in Benin, in urban and rural areas, revealed a mean dietary intake of 4.4 g/24 h of sodium and 1.8 g/24 h of potassium, which are in line with findings from the present study [40].

In a systematic review including data from several countries worldwide, mean sodium intake was always lower in women, with the difference between sexes ranging from 8.9% in South Asia to 10.7% in Western Europe [39]. In the latter study, estimated sodium intakes ranged from 2.18 g/day in Eastern Sub-Saharan Africa to 4.80 g/day in Asian regions [39]. Despite the fact that in our study, similarly to the situation described in other Sub-Saharan African countries [23], no significant differences between sexes were observed in sodium excretion, women presented a higher proportion of urinary sodium to potassium ratio than men. This finding may, at least partially, be explained by the reported significantly higher consumption of sugars, preserves and confectionery by women, as some foods in this group (e.g., cakes, biscuits and cookies) can be important sources of sodium and mostly are low in potassium. In addition, although not statistically significant, women, compared to men, reported a higher consumption of meat products which are also frequently high in sodium.

In our study, discretionary salt was the leading main source of sodium intake, as observed in other studies conducted in different countries [41,42] including South Africa [43], Japan and China [11,44]. Interestingly, besides the use of salt added at the table (35% of the participants) and during cooking (96% of the participants), using stock powder when cooking or adding it to prepared food and salads was shown to be frequent in the present sample of the Mozambican population (70% of the participants). As such, stock powder may be an important source of sodium intake by the Mozambican population, since many people probably do not look at labels and are not aware of sodium contents in these products, using them as additional seasoning. On the contrary, in European and North American countries sodium intake is dominated by sodium added by industry in processed/ultra-processed foods [45]. Yet an increase in the consumption of ultra-processed foods [46] may also be expected in Mozambique, along with globalization. 

In addition to high sodium intake, a low intake of potassium, which is inversely related to blood pressure and to the risk of stroke [47], was also observed. Our data on urinary potassium excretion was well below the lower limit recommended by the WHO, which reflects the low consumption of potassium dietary sources such as fruit, vegetables and pulses. 

The Na/K ratio was also calculated. In addition to being considered a stronger metric for the relation of sodium and blood pressure [16] than either sodium or potassium alone, the ratio may be an indicator of correction for completeness and correlated measurement errors that can occur during the 24-h period of urine collection [18,48]. Furthermore, since this ratio is independent of the total energy intake, unlike sodium and potassium intakes, which are strongly related to energy intake, it is a better index of sodium and potassium intake [49]. Additionally, as revealed by data from the INTERSALT study, there is a higher correlation of casual urinary Na/K ratio with 24-h urinary Na/K ratio than the correlation of casual urinary sodium or potassium and creatinine with 24-h urinary excretion of sodium or potassium, respectively. As such, the estimation of Na/K ratio in casual (“spot”) urine may represent an alternative to the estimation in 24-h urine at a population level and, also, with repeated measurements, for individuals [50].

Higher sodium/potassium ratios are associated with higher blood pressure values. As such, monitoring this ratio over time can contribute to identify populations going through industrialization of diet and at high risk for nutrition-related chronic diseases [51] and increased risk of cardiovascular diseases [52]. We observed a mean sodium/potassium ratio far above the 1:1 ratio suggested by the WHO, which is considered beneficial for health [53]. Our results are consistent with the ones recently published about sodium and potassium intake in South Africa where 77% of the population exceeded the daily recommendation of 5 g salt, 93% of the population did not meet the potassium recommendations and median sodium to potassium ratio was 3.5, which is lower than the mean of our observations [54].

The 24-h urine collection, the major strength of our study, is considered to be the gold standard to assess sodium intake [41,55,56,57,58] since 90% of ingested sodium is excreted in the urine [57]. Besides rigorous validation through urinary creatinine excretion, which minimizes bias due to under- or over-collection, more than a single 24-h urine collection should have been obtained from each participant to decrease daily variability. The misclassification of the levels of sodium excretion in a one day only collection is expected to reflect mostly random error. However, the differences between the dietary intake of sodium in the days when participants collected the urine in relation with their usual intake, either random or due to real changes in intakes induced by the participation in the study, would be expectedly lower if urine collection covered a greater number of days. Nevertheless, the high levels of sodium excretion observed in our study are likely to be conservative estimates, as a Hawthorne effect-like bias would result in healthier behaviors. 

Other limitations of the present study must be discussed. It was based on a non-representative sample, which limits the extrapolation of the obtained estimates to the overall Mozambican population. Although all the participants of the present study were from an urban setting, and employed, and around 50% had at least seven years of education, which could compromise the external validity of the results, inferences to the general population of Maputo may still be possible; in fact, according to the 2008/09 third national family budget survey [59], around 45% of the Mozambican population in the age group of 40 to 49 years had some degree of education, which is progressively increasing in the country, and the city of Maputo presents the best literacy indicators in the country. The prevalence of overweight and obesity in this sample suggest that the participants evaluated are similar to the population from Maputo city regarding this characteristics; a nationally representative survey conducted in 2005 found that one fifth of Mozambican adult population was overweight or obese, with a higher prevalence in women, urban areas and more literate people. The differences in energy intake between sexes were not statistically significant (2479 ± 852 Kcal in women and 2682 ± 1048 Kcal in men, *p* = 0.347), which may reflect limited statistical power, despite men presenting a greater intake. 

The relatively small sample size also limits the precision of the estimates, but it does not compromise the validity of our findings. Also of note, the collection of urinary samples occurred during the warmer months of the year in Mozambique, when it is expected a lower proportion of urinary excretion of the ingested sodium [60]. Accordingly, the present estimates are expectedly lower than if the study had been conducted during the colder season. 

The use of a 24-h dietary recall may also be associated with recall bias, although the short recall period (previous 24 h) allied to the use of memory aids (a photographic book and household measures) may have contributed to minimize it. Social desirability bias may also be present, although the interviewers were trained to help the participants in the process and instructed not to be judgmental. Additionally, underreporting of sodium consumption is commonly seen due to the correlation of sodium intake and total energy intake, which is underreported in dietary recall studies, particularly by women and overweight or obese people [57]. The “subtraction” method used to calculate discretionary sodium is not the gold standard recommendation and as some limitations due mainly to the difficulty in analyzing the sodium contents in the diverse foods. Even so, it represents an acceptable and more accessible way of obtaining discretionary salt intake [61]. 

Also of note, inferences on potassium intake based only in urinary excretions may be misleading as urinary measured potassium may not be representative of the dietary values, due to extra-renal losses, particularly fecal [62]. In the present study, analysis of 24-h dietary recall measures, which has been reported as highly correlated to actual potassium intake [62], showed mean (SD) potassium intake of 3097 (1222) mg for women and 3226 (1754) mg for men (*p* = 0.705), which are higher than the urinary measures. Therefore the compliance with the potassium recommendations were also higher when using the 24-h recall (35.9% in women and 34.9% in men). Accordingly, the mean (SD) ratios were lower than the estimates from urinary excretion, with Na/K of 3.0 (2.4) for women and 2.6 (1.8) for men (*p* = 0.437). Thus, we cannot rule out the possibility that, by using 24-h urinary data, the potassium intake was underestimated in the present study and thus the Na/K ratio may be overestimated. Even so, the Na/K ratio, either by urinary or dietary estimation, was above the recommended by WHO, indication of higher sodium and lower potassium intake in this sample of Mozambican population.

Sodium reduction is considered to be cost effective and one of the top 10 “best buys” interventions for preventing non-communicable diseases (NCDs) [63,64]. It was shown that a reduction of 2400 mg/day in sodium intake predicts a decrease of 5.8 mm Hg in systolic blood pressure after adjustment for age, ethnic group, and blood pressure status [65], which is expected to decrease stroke mortality, ischemic heart disease and other vascular diseases [66]. It is also important to note that the effects of sodium reduction on blood pressure tend to be greater in black people and in hypertensive subjects, which would be of great importance in Mozambique given the high proportion of hypertensive subjects not controlled [67].

In the WHO *Global Action Plan for the Prevention and Control of Non-Communicable Diseases 2013–2020*, one of the key target is to make a 30% relative reduction of mean sodium intake at the population level [68]. In Sub-Saharan countries, under epidemiological and nutritional transitions, this is particularly relevant, since it is expected that there will be a growth of globalization, which is frequently associated with dietary changes including the increase of sodium-rich and potassium-poor foods [69]. 

In a very recent systematic review about salt reduction initiatives around the world it was shown that the Eastern Mediterranean, South-East Asia and Africa are the three regions with the least salt reduction activity and where the NCDs are projected to increase the most [70]. Implementing a salt reduction program, such as the successful one in the United Kingdom (15% reduction in the average salt intake) and already followed by other countries, namely the United States, Canada and Australia, would expectedly represent an important step towards a healthier population and fewer socioeconomic losses [71]. Interventions on salt intake reduction in the Sub-Saharan Africa region were applied in South Africa (through legislation to make the food industry reduce the salt content of selected products) and in Mauritius (through salt reduction in bread) [42].

Our results present a first glimpse on the sodium and potassium consumption in Mozambique. Estimates in samples that are representative of the general population will be needed to confirm the findings from the present study in order to better identify population targets to prioritize interventions. 

The sample of the Mozambican population evaluated in the present study revealed a high consumption on sodium, high Na/K ratio, and low potassium consumption in relation to the WHO recommendations. With the already high prevalence of hypertension in the country, future NCD prevention strategies should emphasize measures for population salt consumption control and enhancement of potassium intake. Besides consumer education, incentives for agricultural production of beans, nuts, fruits and vegetables, which are important food sources of potassium, the promotion of community and school gardens, as well as improvement of the transport network to facilitate the access of those foods, are measures that would promote an increase in potassium intake. According to a recently published revision of intervention strategies for reduction of salt consumption, although strategies involving multiple components, both upstream (regulatory and fiscal interventions, food labelling and media campaigns and population-wide policies such as mandatory reformulation) and downstream interventions (individually focused interventions like dietary counselling for individuals, worksites or communities), achieved the biggest population level reductions in salt consumption; the greater effects in population-wide salt consumption were observed with upstream, population-level strategies [72]. In a low-income country as Mozambique, where deprived groups more often consume foods high in salt, sugar and fat, inequalities may be widened by downstream interventions focused on individuals; upstream structural interventions are probably more indicated, as they may reduce inequalities and can be rapid, cost-effective and cost-saving. Further study on political feasibility and stakeholder influence are needed in order to set targets for population salt and potassium intake and develop a strategy, involving different stakeholders, namely the government and the food industries, to reduce sodium and increase potassium intakes. 

## 5. Conclusions

In this convenience sample of Maputo inhabitants, less than one out of 10 participants met the recommended levels of sodium and potassium intakes, and the sodium-to-potassium ratio was far higher than the level recommended by WHO. Sodium from salt and stock powder added to culinary preparations was the most reported contributor for the total intake. This results suggest that population-level measures to modify the current patterns of consumption of sodium and potassium in the country are warranted towards the prevention of the farseeing additional burden to the health system. The main sources of sodium intake uncovered by this study should be further explored at a national level, if possible, to clarify their potential as targets for tailored interventions aiming to decrease salt intake towards the prevention of NCDs in Mozambique.

## Figures and Tables

**Table 1:** Characteristics of the study sample, overall and by sex.

	Total (*n* = 82)	Women (*n* = 39)	Men (*n* = 43)	*p*
Age (years), mean (SD)	39.9 (9.6)	41.8 (10.5)	38.1 (8.5)	0.082
Education level, *n* (%)				
Primary school not completed	17 (20.7)	11 (28.2)	6 (14.0)	0.331
Primary school completed	43 (52.4)	18 (46.2)	25 (58.1)	
Secondary school completed	18 (22.0)	9 (23.1)	9 (20.9)	
Post-secondary school	4 (4.9)	1 (2.6)	3 (7.0)	
BMI (kg/m^2^) , mean (SD)	26.7 (5.8)	29.6 (6.4)	24.1 (3.7)	<0.001
BMI categories, *n* (%)				
Thinness	1 (1.2)	0	1 (2.3)	0.001
Normal weight	39 (47.6)	13 (33.3)	26 (60.5)	
Overweight	25 (30.5)	11 (28.2)	14 (32.6)	
Obesity	17 (20.7)	15 (38.5)	2 (4.7)	
Waist circumference (cm), mean (SD)	89.2 (14.7)	93.8 (14.7)	85.0 (13.5)	0.007
Abdominal obesity, *n* (%)	27 (32.9)	24 (61.5)	3 (7.0)	<0.001

Standard deviation (SD); Body mass index (BMI).

**Table 2:** Dietary intake of the studied sample, overall and by sex.

Dietary Intake (g/Day) *	*n* ^†^	Total	*n* ^†^	Women	*n* ^†^	Men	*p*
Cereal and cereal products	82	360 (63,1507)	39	322 (103, 1081)	43	407 (63, 1507)	0.072
Wheat Bread	66	200 (60, 700)	31	150 (60, 600)	35	300 (100, 700)	0.018
Rice	69	107 (27, 320)	33	107 (27, 320)	36	107 (27, 320)	0.804
Beans	26	94 (31, 250)	10	94 (31, 172)	16	94 (47, 250)	0.336
Meat products	51	125 (16, 500)	23	125 (16, 500)	28	89 (40, 375)	0.161
Fish and seafood dishes	38	94 (25, 375)	17	94 (63, 172)	21	94 (25, 375)	0.728
Eggs	23	55 (28, 110)	14	55 (28, 55)	9	55 (55, 110)	0.369
Milk and milk products	21	44 (8, 430)	6	47 (22, 430)	15	44 (8, 300)	0.622
Vegetables	77	94 (10, 361)	36	84 (13, 361)	41	102 (10, 294)	0.709
Fruits	49	188 (34, 1016)	25	188 (47, 1016)	24	188 (34, 958)	0.763
Oils and fats	79	9 (1, 105)	36	9 (1, 27)	43	9 (1, 105)	0.694
Sugars, preserves and confectionery	72	54 (1, 1837)	37	166 (1, 784)	35	21 (4, 1837)	0.016
Other foods	58	101 (9, 318)	27	101 (31, 203)	31	101 (9, 318)	0.656
Peanut	39	94 (34, 203)	19	94 (34, 205)	20	96 (45, 169)	0.857

* Results are presented as median (minimum, maximum); ^†^ Corresponds to the number of participants consuming each food item of food items from each group.

**Table 3:** Urinary data on sodium and potassium excretion, overall and by sex.

	Total	Women	Men	*p*
Sodium (mg/day), mean (SD)	4220 (1830)	4538 (2033)	3931 (1593)	0.135
Salt (g/day), mean (SD)	10.6 (4.6)	11.3 (5.1)	9.8 (4.0)	0.135
Compliance with recommendations, *n* (%) *	7 (8.5)	2 (5.1)	5 (11.6)	0.455
Potassium (mg/day), mean (SD)	1909 (778)	1841 (780)	1970 (779)	0.537
Compliance with recommendations, *n* (%) ^†^	3 (3.7)	1 (2.6)	2 (4.7)	0.537
Ratio Na/K, mean (SD) ^¥^	4.2 (2.4)	4.7 (2.6)	3.7 (2.1)	0.061

SD—standard deviation; * The upper limit for sodium intake recommended by the World Health Organization (WHO) is 2000 mg per day; ^†^ The WHO recommends a minimum daily intake of 3510 mg of potassium per day; ^¥^ Molar Ratio Na/K estimated taking into account the molar weight of sodium (23 g/mol) and potassium (39 g/mol).

## References

[1] Suckling R.J., He F.J., Macgregor G.A. (2010). Altered dietary salt intake for preventing and treating diabetic kidney disease. Cochrane Database Syst. Rev..

[2] Poggio R., Gutierrez L., Matta M.G., Elorriaga N., Irazola V., Rubinstein A. (2015). Daily sodium consumption and cvd mortality in the general population: Systematic review and meta-analysis of prospective studies. Public Health Nutr..

[3] McMahon E.J., Campbell K.L., Bauer J.D., Mudge D.W. (2015). Altered dietary salt intake for people with chronic kidney disease. Cochrane Database Syst. Rev..

[4] World Health Organization (WHO) Guideline: Sodium Intake for Adults and Children. http://apps.who.int/iris/bitstream/10665/77985/1/9789241504836_eng.pdf?ua=1&ua=1.

[5] Brown I.J., Tzoulaki I., Candeias V., Elliott P. (2009). Salt intakes around the world: Implications for public health. Int. J. Epidemiol..

[6] Whelton P.K., He J., Cutler J.A., Brancati F.L., Appel L.J., Follmann D., Klag M.J. (1997). Effects of oral potassium on blood pressure: Meta-analysis of randomized controlled clinical trials. JAMA.

[7] Geleijnse J.M., Kok F.J., Grobbee D.E. (2003). Blood pressure response to changes in sodium and potassium intake: A metaregression analysis of randomised trials. J. Hum. Hypertens..

[8] Aaron K.J., Sanders P.W. (2013). Role of dietary salt and potassium intake in cardiovascular health and disease: A review of the evidence. Mayo Clinic proceedings. Mayo Clin..

[9] Morris R.C., Schmidlin O., Frassetto L.A., Sebastian A. (2006). Relationship and interaction between sodium and potassium. J. Am. Coll. Nutr..

[10] Geleijnse J.M., Witteman J.C.M., Stijnen T., Kloos M.W., Hofman A., Grobbee D.E. (2007). Sodium and potassium intake and risk of cardiovascular events and all-cause mortality: The Rotterdam study. Eur. J. Epidemiol..

[11] Stamler J., Rose G., Stamler R., Elliott P., Dyer A., Marmot M. (1989). Intersalt study findings. Public health and medical care implications. Hypertension.

[12] Whelton P.K. (2014). Sodium, potassium, blood pressure, and cardiovascular disease in humans. Curr. Hypertens. Rep..

[13] Kotchen T.A., Kotchen J.M., Shils M.E., Shike M., Ross A.C., Caballero B., Cousins R.J. (2006). Nutrition, diet, and hypertension. Modern Nutrition in Health and Disease.

[14] Thornton S.N. (2013). Salt in health and disease—A delicate balance. N. Engl. J. Med..

[15] World Health Organization (WHO) Guideline: Potassium Intake for Adults and Children. http://www.who.int/nutrition/publications/guidelines/potassium_intake/en/.

[16] Rose G., Stamler J., Stamler R., Elliott P., Marmot M., Pyorala K., Kesteloot H., Joossens J., Hansson L., Mancia G. (1988). Intersalt: An international study of electrolyte excretion and blood pressure. Results for 24 hour urinary sodium and potassium excretion. Intersalt cooperative research group. Br. Med. J..

[17] Umesawa M., Iso H., Date C., Yamamoto A., Toyoshima H., Watanabe Y., Kikuchi S., Koizumi A., Kondo T., Inaba Y. (2008). Relations between dietary sodium and potassium intakes and mortality from cardiovascular disease: The japan collaborative cohort study for evaluation of cancer risks. Am. J. Clin. Nutr..

[18] Cook N.R., Obarzanek E., Cutler J.A., Buring J.E., Rexrode K.M., Kumanyika S.K., Appel L.J., Whelton P.K., Trials of Hypertension Prevention Collaborative Research Group (2009). Joint effects of sodium and potassium intake on subsequent cardiovascular disease: The trials of hypertension prevention (TOHP) follow-up study. Arch. Intern. Med..

[19] Cook N.R., Kumanyika S.K., Cutler J.A. (1998). Effect of change in sodium excretion on change in blood pressure corrected for measurement error: The trials of hypertension prevention, phase I. Am. J. Epidemiol..

[20] Khaw K.T., Barrett-Connor E. (1988). The association between blood pressure, age, and dietary sodium and potassium: A population study. Circulation.

[21] Perez V., Chang E.T. (2014). Sodium-to-potassium ratio and blood pressure, hypertension, and related factors. Adv. Nutr..

[22] Carvalho J.J., Baruzzi R.G., Howard P.F., Poulter N., Alpers M.P., Franco L.J., Marcopito L.F., Spooner V.J., Dyer A.R., Elliott P. (1989). Blood pressure in four remote populations in the INTERSALT Study. Hypertension.

[23] Oyebode O., Oti S., Chen Y.-F., Lilford R.J. (2016). Salt intakes in Sub-Saharan Africa: A systematic review and meta-regression. Popul. Health Metr..

[24] Damasceno A., Azevedo A., Silva-Matos C., Prista A., Diogo D., Lunet N. (2009). Hypertension prevalence, awareness, treatment, and control in Mozambique: Urban/rural gap during epidemiological transition. Hypertension.

[25] Damasceno A., Gomes J., Azevedo A., Carrilho C., Lobo V., Lopes H., Madede T., Pravinrai P., Silva-Matos C., Jalla S. (2010). An epidemiological study of stroke hospitalizations in Maputo, Mozambique: A high burden of disease in a resource-poor country. Stroke.

[26] Dgedge M., Novoa A., Macassa G., Sacarlal J., Black J., Michaud C., Cliff J. (2001). The burden of disease in maputo city, Mozambique: Registered and autopsied deaths in 1994. Bull. World Health Organ..

[27] The World Bank Urban Population (% of Total). United Nations, World Urbanization Prospects. http://data.worldbank.org/indicator/SP.URB.TOTL.IN.ZS.

[28] Popkin B.M. (2011). Contemporary nutritional transition: Determinants of diet and its impact on body composition. Proc. Nutr. Soc..

[29] Silva V., Santos S., Novela C., Padrão P., Moreira P., Lunet N., Damasceno A. In Some observations on food consumption and culinary practices in Maputo, Mozambique. Proceedings of the 8th International Conference on Culinary Arts and Sciences: Global, National and Local Perspectives.

[30] Gomes A., Damasceno A., Azevedo A., Prista A., Silva-Matos C., Saranga S., Lunet N. (2010). Body mass index and waist circumference in Mozambique: Urban/rural gap during epidemiological transition. Obes. Rev..

[31] Gomes J., Damasceno A., Carrilho C., Lobo V., Lopes H., Madede T., Pravinrai P., Silva-Matos C., Diogo D., Azevedo A. (2013). Determinants of early case-fatality among stroke patients in Maputo, Mozambique and impact of in-hospital complications. Int. J. Stroke.

[32] WHO Regional Office for Europe (1984). Estimation of Sodium Intake and Output: Review of Methods and Recommendations for Epidemiological Studies.

[33] Korkalo L., Hauta-alus H., Mutanen M. (2011). Food Composition Tables for Mozambique.

[34] Giuntini E., Lajolo F., Menezes E. (2006). Brazilian food composition table TBCA-USP (versions 3 and 4) in the international context. Arch. Latinoam. Nutr..

[35] Lohman T., Roache A., Martorell R. (1992). Anthropometric standardization reference manual. Med. Sci. Sports Exerc..

[36] World Health Organization (WHO) Body Mass Index-BMI. http://www.euro.who.int/en/health-topics/disease-prevention/nutrition/a-healthy-lifestyle/body-mass-index-bmi.

[37] Expert Panel on Detection Evaluation and Treatment of High Blood Cholesterol in Adults (2001). Executive summary of the third report of the national cholesterol education program (NCEP) expert panel on detection, evaluation, and treatment of high blood cholesterol in adults (adult treatment panel Ш). JAMA.

[38] World Health Organization (WHO) Global Strategy on Diet, Physical Activity and Health. Promoting Fruit and Vegetable Consumption around the World. http://www.who.int/dietphysicalactivity/fruit/en/.

[39] Powles J., Fahimi S., Micha R., Khatibzadeh S., Shi P., Ezzati M., Engell R.E., Lim S.S., Danaei G., Mozaffarian D. (2013). Global, regional and national sodium intakes in 1990 and 2010: A systematic analysis of 24 h urinary sodium excretion and dietary surveys worldwide. BMJ Open.

[40] Mizéhoun-Adissoda C., Houinato D., Houehanou C., Chianea T., Dalmay F., Bigot A., Aboyans V., Preux P.-M., Bovet P., Desport J.-C. (2017). Dietary sodium and potassium intakes: Data from urban and rural areas. Nutrition.

[41] Xu J., Wang M., Chen Y., Zhen B., Li J., Luan W., Ning F., Liu H., Ma J., Ma G. (2014). Estimation of salt intake by 24-h urinary sodium excretion: A cross-sectional study in Yantai, china. BMC Public Health.

[42] Sookram C., Munodawafa D., Phori P.M., Varenne B., Alisalad A. (2015). Who’s supported interventions on salt intake reduction in the Sub-Saharan Africa region. Cardiovasc. Diagn. Ther..

[43] Charlton K.E., Steyn K., Levitt N.S., Zulu J.V., Jonathan D., Veldman F.J., Nel J.H. (2005). Diet and blood pressure in south Africa: Intake of foods containing sodium, potassium, calcium, and magnesium in three ethnic groups. Nutrition.

[44] Stamler J., Elliott P., Dennis B., Dyer A.R., Kesteloot H., Liu K., Ueshima H., Zhou B.F. (2003). Intermap: Background, aims, design, methods, and descriptive statistics (nondietary). J. Hum. Hypertens..

[45] Ni Mhurchu C., Capelin C., Dunford E.K., Webster J.L., Neal B.C., Jebb S.A. (2011). Sodium content of processed foods in the united kingdom: Analysis of 44,000 foods purchased by 21,000 households. Am. J. Clin. Nutr..

[46] Monteiro C.A., Levy R.B., Claro R.M., de Castro I.R., Cannon G. (2011). Increasing consumption of ultra-processed foods and likely impact on human health: Evidence from Brazil. Public Health Nutr..

[47] O’Donnell M., Mente A., Rangarajan S., McQueen M.J., Wang X., Liu L., Yan H., Lee S.F., Mony P., Devanath A. (2014). Urinary sodium and potassium excretion, mortality, and cardiovascular events. N. Engl. J. Med..

[48] Espeland M.A., Kumanyika S., Wilson A.C., Reboussin D.M., Easter L., Self M., Robertson J., Brown W.M., McFarlane M. (2001). Statistical issues in analyzing 24-h dietary recall and 24-h urine collection data for sodium and potassium intakes. Am. J. Epidemiol..

[49] Cobb L.K., Anderson C.A., Elliott P., Hu F.B., Liu K., Neaton J.D., Whelton P.K., Woodward M., Appel L.J. (2014). Methodological issues in cohort studies that relate sodium intake to cardiovascular disease outcomes: A science advisory from the American heart association. Circulation.

[50] Iwahori T., Miura K., Ueshima H., Chan Q., Dyer A.R., Elliott P., Stamler J., INTERSALT Research Group (2016). Estimating 24-h urinary sodium/potassium ratio from casual (‘spot’) urinary sodium/potassium ratio: The INTERSALT Study. Int. J. Epidemiol..

[51] Hedayati S.S., Minhajuddin A.T., Ijaz A., Moe O.W., Elsayed E.F., Reilly R.F., Huang C.L. (2012). Association of urinary sodium/potassium ratio with blood pressure: Sex and racial differences. Clin. J. Am. Soc. Nephrol..

[52] Yang Q., Liu T., Kuklina E.V., Flanders W.D., Hong Y., Gillespie C., Chang M.H., Gwinn M., Dowling N., Khoury M.J. (2011). Sodium and potassium intake and mortality among us adults: Prospective data from the third national health and nutrition examination survey. Arch. Intern. Med..

[53] World Health Organization (WHO) Diet, Nutrition and the Prevention of Chronic Diseases. http://www.who.int/dietphysicalactivity/publications/trs916/en/.

[54] Swanepoel B., Schutte A.E., Cockeran M., Steyn K., Wentzel-Viljoen E. (2016). Sodium and potassium intake in south africa: An evaluation of 24-h urine collections in a white, black, and Indian population. J. Am. Soc. Hypertens..

[55] Ribič C.H., Zakotnik J.M., Vertnik L., Vegnuti M., Cappuccio F.P. (2010). Salt intake of the Slovene population assessed by 24 h urinary sodium excretion. Public Health Nutr..

[56] Aparicio A., Rodriguez-Rodriguez E., Cuadrado-Soto E., Navia B., Lopez-Sobaler A.M., Ortega R.M. (2017). Estimation of salt intake assessed by urinary excretion of sodium over 24 h in Spanish subjects aged 7–11 years. Eur. J. Nutr..

[57] McLean R.M. (2014). Measuring population sodium intake: A review of methods. Nutrients.

[58] Zhang J., Yan L., Tang J., Ma J., Guo X., Zhao W., Zhang X., Li J., Chu J., Bi Z. (2014). Estimating daily salt intake based on 24 h urinary sodium excretion in adults aged 18–69 years in Shandong, china. BMJ Open.

[59] Khossa D., Macaringue F., Nassabe J., Ismael M., Nhantumbo N., Mandlate T. Estatísticas e Indicadores Sociais, 2008–2010. Instituto Nacional de Estatística. Direcção de Estatísticas Demográficas Vitais e Sociais. www.ine.gov.mz.

[60] Conkle J., van der Haar F. (2017). The use and interpretation of sodium concentrations in casual (spot) urine collections for population surveillance and partitioning of dietary iodine intake sources. Nutrients.

[61] Pan American Health Organization (2011). Regional Expert Group for Cardiovascular Disease. Prevention through Population-Wide Dietary Salt Reduction. http://www2.paho.org/hq/index.php?option=com_docman&task=doc_view&Itemid=270&gid=21491&lang=en.

[62] Iwahori T., Miura K., Ueshima H. (2017). Time to consider use of the sodium-to-potassium ratio for practical sodium reduction and potassium increase. Nutrients.

[63] Zarocostas J. (2011). Who lists “best buys” for cutting deaths from non-communicable disease. BMJ.

[64] Eyles H., Shields E., Webster J. (2016). Achieving the who sodium target: Estimation of reductions required in the sodium content of packaged foods and other sources of dietary sodium. Am. J. Clin. Nutr..

[65] He F.J., Li J., MacGregor G.A. (2013). Effect of longer term modest salt reduction on blood pressure: Cochrane systematic review and meta-analysis of randomised trials. BMJ.

[66] Ettehad D., Emdin C.A., Kiran A., Anderson S.G., Callender T., Emberson J., Chalmers J., Rodgers A., Rahimi K. (2016). Blood pressure lowering for prevention of cardiovascular disease and death: A systematic review and meta-analysis. Lancet.

[67] Appel L.J., Brands M.W., Daniels S.R., Karanja N., Elmer P.J., Sacks F.M. (2006). Dietary approaches to prevent and treat hypertension: A scientific statement from the American heart association. Hypertension.

[68] World Health Organization (WHO) Global Action Plan for the Prevention and Control of NCDs 2013–2020. http://www.who.int/nmh/events/ncd_action_plan/en/.

[69] Popkin B.M. (2004). The nutrition transition: An overview of world patterns of change. Nutr. Rev..

[70] Trieu K., Neal B., Hawkes C., Dunford E., Campbell N., Rodriguez-Fernandez R., Legetic B., McLaren L., Barberio A., Webster J. (2015). Salt reduction initiatives around the world-A systematic review of progress towards the global target. PLoS ONE.

[71] He F.J., Brinsden H.C., MacGregor G.A. (2014). Salt reduction in the United Kingdom: A successful experiment in public health. J. Hum. Hypertens..

[72] Hyseni L., Elliot-Green A., Lloyd-Williams F., Kypridemos C., O’Flaherty M., McGill R., Orton L., Bromley H., Cappuccio F.P., Capewell S. (2017). Systematic review of dietary salt reduction policies: Evidence for an effectiveness hierarchy?. PLoS ONE.

